# Association Between the *LZTFL1* rs11385942 Polymorphism and COVID-19 Severity in Colombian Population

**DOI:** 10.3389/fmed.2022.910098

**Published:** 2022-06-20

**Authors:** Mariana Angulo-Aguado, David Corredor-Orlandelli, Juan Camilo Carrillo-Martínez, Mónica Gonzalez-Cornejo, Eliana Pineda-Mateus, Carolina Rojas, Paula Triana-Fonseca, Nora Constanza Contreras Bravo, Adrien Morel, Katherine Parra Abaunza, Carlos M. Restrepo, Dora Janeth Fonseca-Mendoza, Oscar Ortega-Recalde

**Affiliations:** ^1^Center for Research in Genetics and Genomics – CIGGUR, GENIUROS Research Group, School of Medicine and Health Sciences, Universidad Del Rosario, Bogotá, Colombia; ^2^Department of Molecular Diagnosis, Genética Molecular de Colombia SAS, Bogotá, Colombia; ^3^Hospital Universitario Mayor – Méderi – Universidad del Rosario, Bogotá, Colombia

**Keywords:** *LZTFL1*, *ACE*, *ACE2*, host genetics, infection severity, COVID-19

## Abstract

Genetic and non-genetic factors are responsible for the high interindividual variability in the response to SARS-CoV-2. Although numerous genetic polymorphisms have been identified as risk factors for severe COVID-19, these remain understudied in Latin-American populations. This study evaluated the association of non-genetic factors and three polymorphisms: *ACE* rs4646994, *ACE2* rs2285666, and *LZTFL1* rs11385942, with COVID severity and long-term symptoms by using a case-control design. The control group was composed of asymptomatic/mild cases (*n* = 61) recruited from a private laboratory, while the case group was composed of severe/critical patients (*n* = 63) hospitalized in the Hospital Universitario Mayor-Méderi, both institutions located in Bogotá, Colombia. Clinical follow up and exhaustive revision of medical records allowed us to assess non-genetic factors. Genotypification of the polymorphism of interest was performed by amplicon size analysis and Sanger sequencing. In agreement with previous reports, we found a statistically significant association between age, male sex, and comorbidities, such as hypertension and type 2 diabetes mellitus (T2DM), and worst outcomes. We identified the polymorphism *LZTFL1* rs11385942 as an important risk factor for hospitalization (*p* < 0.01; OR = 5.73; 95% CI = 1.2–26.5, under the allelic test). Furthermore, long-term symptoms were common among the studied population and associated with disease severity. No association between the polymorphisms examined and long-term symptoms was found. Comparison of allelic frequencies with other populations revealed significant differences for the three polymorphisms investigated. Finally, we used the statistically significant genetic and non-genetic variables to develop a predictive logistic regression model, which was implemented in a Shiny web application. Model discrimination was assessed using the area under the receiver operating characteristic curve (AUC = 0.86; 95% confidence interval 0.79–0.93). These results suggest that *LZTFL1* rs11385942 may be a potential biomarker for COVID-19 severity in addition to conventional non-genetic risk factors. A better understanding of the impact of these genetic risk factors may be useful to prioritize high-risk individuals and decrease the morbimortality caused by SARS-CoV2 and future pandemics.

## Introduction

SARS-CoV-2 (Severe acute respiratory syndrome coronavirus 2) is a novel coronavirus, first identified in China in late December 2019 (1). The disease caused by this virus, named COVID-19, rapidly spread across the globe being declared a pandemic by the WHO in March 2021 (2). Up to the first week of March 2022, more than 450million confirmed cases and 6 million deaths were reported worldwide, from which ~6million confirmed cases and 139.000 deaths occurred in Colombia (3, 4). The clinical course and severity of COVID-19 disease are highly variable among individuals, ranging from asymptomatic cases to severe respiratory failure and death (5).

Different clinical risk factors, including aging, male sex and comorbidities such as cardiovascular disease, hypertension, diabetes mellitus, chronic obstructive pulmonary lung disease, immunosuppression and obesity have been linked to more severe courses of COVID-19 (6, 7). Importantly, numerous studies have shown that host genetic factors also play a critical role in SARS-CoV-2 disease progression and severity (8–10). Early works suggested a potential role of genes related to the renin-angiotensin-aldosterone system (RAAS) (*ACE1* and *ACE2*), the ABO blood group system and the human leukocyte antigen (HLA) (11–13). The RAAS pathway is a physiological system that plays an important role in the homeostatic control of blood pressure and body water-electrolyte balance (14). Angiotensin I converting enzyme and angiotensin converting enzyme 2, coded by the genes *ACE* and *ACE2*, respectively, are critical regulators of this pathway and may also contribute to multiple organ injuries in COVID-19. In lung vascular endothelium, ACE catalyzes Angiotensin I conversion into Angiotensin II, an active peptide that promotes vasoconstriction, inflammation and thrombosis (15). Conversely, ACE2 converts Angiotensin II into angiotensin-(1–7), molecules that counteract the effects of Angiotensin II, including vasodilatation and vascular protection (16). Polymorphisms that increase *ACE* expression have been associated with more severe COVID-19 infections. The *ACE* insertion(Ins)/deletion(Del) polymorphism (rs4646994) is of particular interest as the resulting decrease in ACE activity has been linked to a protective effect in Ins allele carriers (17). Moreover, ACE2 has a dual role as the SARS-CoV-2 receptor, allowing virus internalization, and as RAAS regulator, catalyzing angiotensin II degradation (16, 18). Whole exome studies (WES) have identified more than 30 variants in the *ACE2* gene, potentially interfering with protein structure, stabilization and expression, and contributing to the high interindividual variability and susceptibility to COVID-19 (19). Among these variants, NM_001371415.1:c.439+4G>A (rs2285666) polymorphism is related to an increase of 50% of ACE2 expression, compared to wild-type G/G genotype carriers, and decreases the risk of severe SARS-CoV2 infection (20). In addition, two large genome-wide association studies, oriented to find genetic susceptibly locus, identified an association signal at chromosome 3p21.31 (rs11385942 and rs10490770) as the one with the most significant association with respiratory failure and mechanical ventilation requirement amongst severe COVID-19 patients (21, 22). This locus contains several genes related to cell signaling and solute transportation, including *CCR9, CXCR6, LZTFL1*, and *SLC6A20*. *LZTFL1* gene, the most promising candidate, codifies for a protein involved in the primary cilia function and the immunological synapse between T-cells and antigen-presenting cells (23).

Despite their relevance, genetic host factors related to COVID-19 severity remain understudied in Latin-American populations, limiting their potential use as predictive biomarkers and the development of predictive models. Furthermore, the study of these factors is particularly relevant considering that Latin-American countries have been severely affected by the COVID-19 pandemic. In this study, we performed an ambispective case-control analysis to evaluate the association between non-genetic factors and genetic factors, including the polymorphisms rs4646994 (*ACE*), rs2285666 (*ACE2*), and rs11385942 (*LZTFL1*), and COVID-19 severity and long-term symptoms in Colombian population. The results of this study support a positive association between the *LZTFL1* rs11385942 locus variant and an increased risk of severe SARS-CoV-2 infection. Furthermore, we developed a predictive model integrating non-genetic and genetic factors, potentially useful to identify high-risk individuals and prioritize prevention and mitigation efforts.

## Methods

### Study Population and Sampling

This study enrolled 145 patients between 18 and 60 years with confirmed diagnosis of COVID-19 by positive RT-PCR (reverse transcriptase polymerase chain reaction), antigens or antibodies (IgG and/or IgM for SARS-CoV-2) tests. The control group consisted of 71 patients who were classified as asymptomatic or mild COVID-19, group non-hospitalized. The case group was composed of 74 patients with severe or critical disease, group hospitalized. Subcategorization of the case group was made with patients critically ill who required intensive care unit (ICU), group hospitalized-ICU. Clinical severity was determined according to national guidelines for COVID-19 by the Colombian Health Ministry (24). Cases were recruited among hospitalized patients at the Hospital Universitario Mayor-Méderi (Bogotá, Colombia). Controls were enrolled from a private laboratory (Genética Molecular de Colombia, Bogotá, Colombia). Cases and controls were invited to participate in this study and those who accepted signed an informed consent and underwent buccal swap or peripheral blood sampling. Patients were enrolled between December 2020–July 2021 and all subjects were unvaccinated at the time of recruitment.

The sample size was calculated with a *p* (sample proportion) of 7% according to the minimum allele frequency (MAF) for the allele with the reported lowest frequency, in our case the polymorphism rs11385942, a confidence level of 95% (α = 0.05, z = 1.96), a margin of error (*e*) of 5%, and a population size *N* = 8,000,000 for Bogotá city. Using the formula *n* = N*z*^2*^p(1-*p*)/α^2^(N-1)+z^2^*p(1-p), implemented in the OpenEpi web-tool, we estimated that the minimum sample size was 101 (25). This value was approximated to 145 individuals considering possible clinical follow up lost. Given this is the first study to assess allele frequency for the polymorphisms of interest in Colombian population, MAF were obtained from the GnomAD database for Latino-American individuals (26). This study followed the guidelines of the Declaration of Helsinki and all experimental procedures were approved by the Ethics Committee of Universidad del Rosario (DVO005 1543-CV1334).

### Clinical Data Collection and Follow Up

Data collection and clinical follow up were conducted through phone calls at least 21 days after the diagnosis. Data was obtained through a standardized format that included the following clinical and demographical information: sex, age, blood type, medical history, comorbidities, drugs use, symptoms, long-term symptoms, and any change in disease severity. Furthermore, we performed an exhaustive revision of clinical records of hospitalized patients to validate the information collected previously and verify the clinical classification and severity criteria according to the clinical guidelines mentioned before. One hundred and twenty four patients, 61 cases and 63 controls, completed the clinical follow up and continued in the study.

### DNA Extraction and Genotyping

Total genomic DNA was obtained from buccal swab or blood samples using either the Quick-DNA™ Miniprep Plus Kit (Zymo Research) or the Buccal Swab DNA Kit (Promega). The buccal swab samples were collected in a cotton swab and the blood samples were collected in EDTA tubes, 5mL for patient. Genomic DNA was quantified using a nanodrop spectrophotometer. All samples were aliquoted and stored at 4°C until analysis. Polymerase chain reaction (PCR) was used to amplify and genotype three polymorphisms of interest: *ACE* 289bp ALU Ins/Del (rs4646994), *ACE2* c.439+4G>A (rs2285666), and *LZTFL1* c.323+621dup (rs11385942). Primers were designed using PrimerBlast (27). Primers sequences and PCR conditions are listed in Supplementary Table 1. For *ACE* rs4646994 genotyping, PCR products were run on a 1% agarose gel stained by ethidium bromide and amplicon sizes were used to determine individual genotypes. Fragments obtained were 191 bp for the Del allele and 480 bp for the Ins allele. For *ACE2* rs2285666 and *LZTL1* rs11385942, PCR products were purified and sequenced through Sanger method. Sequences were analyzed with the software Geneious Prime v2021.2 (Biomatters) (28). Genotypes were assigned in batches of 20 samples by two independent researchers. In case the results were in disagreement, a third researcher reassessed the results and a final consensus was achieved. These researchers were blind to the case-control status of the individuals. Genotypification was attempted in 125 individuals, being successful in 124 (99.2%).

### Statistical Analysis and Predictive Model

A bivariate analysis was performed between clinical and demographic variables with the severe COVID-19 outcome (non-hospitalized vs. hospitalized, including UCI and non-UCI patients) or the presence of long-term COVID-19 symptoms using the χ^2^, Mann-Whitney and OR statistics. All the analyses were conducted using this case-control definition unless otherwise stated. Significant thresholds were set as *p* < 0.05, and a 95% confidence interval for the OR. Long-term COVID-19 symptoms were defined as persistent symptoms beyond 3 weeks from initial symptoms onset (29). An extended analysis of long-term symptoms was performed grouping symptoms into the following categories: (1) frequent (fatigue, headache, attention deficit, alopecia, dyspnea), (2) organ system affected (neurological, psychiatric, osteomuscular, respiratory, and cardiovascular), and (3) others including the ones with low sample and literature prevalence (dysphagia, otorhinolaryngological, ophthalmological and cutaneous manifestations) according to Lopez-Leon et al. (30).

Population genetic statistics, including allelic frequencies, genotypic frequencies and Hardy–Weinberg equilibrium (HWE), were calculated using the SNPStats software (31). The deviation of the HWE was established using a χ^2^ goodness-of-fit test with 1° of freedom (df) except for the SNP in *ACE2* rs2285666 located in the X chromosome, for which HWE was determined using the R package “HWadmiX” (32). Allelic frequencies obtained from the study were compared to other populations using the χ^2^ and Fisher's exact test statistics (21, 26, 33–46). *p*-values <0.05 were considered statistically significant.

The bivariate association analysis between genetic polymorphisms and severity outcome or the presence of long-term symptoms was performed with the PLINK software (47). Different genetic models, including allelic, genotypic, dominant and recessive, were assessed with the Cochran-Armitage trend, genotypic (2df), dominant gene action (1df), and recessive gene (1df) tests. In addition, a subgroup analysis between control (non-hospitalized) and ICU-hospitalized patients (*n* = 26) was conducted under the allelic model. The clinical and genetic variables with a significant correlation were used to build a multivariate logistic regression model in order to develop a predictive risk model for severe disease. Different combinations of variables were tested to construct the models, and these were compared using the Akaike Information Criterion (AIC) and the Coefficient of Discrimination D (Tjur's R2) parameters. This last method, Tjur's R2, is used for binomial logistic models and a value approaching 1 indicates that there is a clear separation between the predicted values for the response outcomes (48). For the model construction we evaluated and handled the potentially cofounding and interacting variables. We assessed the variation inflation factor (VIF) to protect our model to be inflated by multicollinearity, all the variables included had a VIF value of 1. Model comparison was assessed by calculating the area under the receiver operating characteristic curve (AUC), direct comparison between the scores obtained from the models, integrated discrimination improvement (IDI) and cross-validation parameters, including concordance, sensitivity, specificity, and net benefit at different cutoff probabilities. Concordance was defined as the correctly estimated outcomes using several cutoff values for the predicted affection probability. The IDI score and cross-validation parameters were calculated with the R packages PredictABEL and rmda, respectively (49, 50). Finally, an open-source and online application was developed for users to easily access and test the model. The predictive model was constructed using R v4.1.2 and the online application was built using the Shiny package for R (51).

## Results

### Clinical and Demographic Data

**Figure 1:**
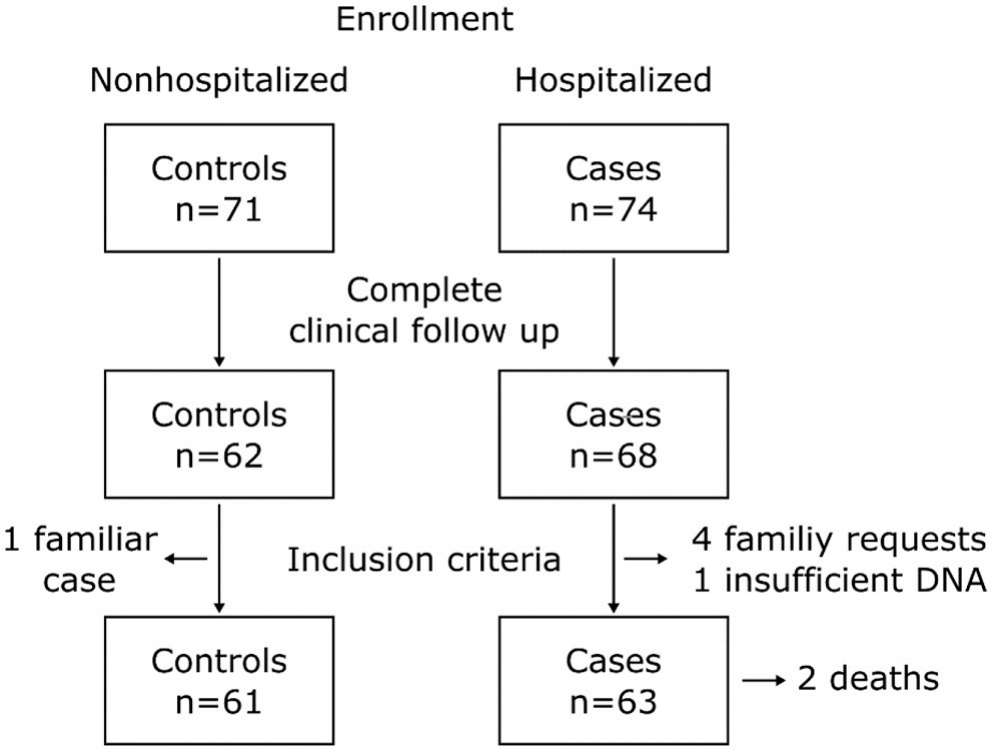
Flowchart of the study participants.

In total 145 patients, 71 controls and 74 cases were enrolled in the study. Nine patients from the control group and six patients from the cases group were excluded from the study by loss to follow up. One control was excluded due to familial relationship, one case was excluded by insufficient DNA and four cases were excluded due to direct request from the family. The final number of patients included was 61 controls and 63 cases. Two patients from the cases group died due to COVID-19 complications; nevertheless, clinical follow up was completed with help of relatives. A summary of the study participants is presented in Figure 1. For the control group, 29.5% (*n* = 18) diagnoses were made by RT-PCR, 63.9% (*n* = 39) by antigen test, and 6.6% (*n* = 4) by antibodies. The sampling methods for this group were 67.2% (*n* = 41) by buccal swabs and 32.8% (*n* = 20) from peripheral blood. For the case group, 98.4% (*n* = 62) diagnoses were made by RT-PCR and 1.6% (*n* = 1) by antigen test and 100% samples were taken from peripheral blood.

Demographic and clinical characteristics of our study population are summarized in Table 1. The mean age for the control group was 36.6 ± 10.8 years and that for the case group was 47.3 ± 9.53 years. Men accounted 42.6% (*n* = 26) of controls and 65% (*n* = 41) of the case group. Among the most common comorbidities in our study population were type 2 diabetes mellitus (T2DM) 11.3% (*n* = 14), hypertension 16.1% (*n* = 20) and obesity 21.8% (*n* = 27). Most patients (56.5%, *n* = 70) presented no comorbidities, 27.4 (*n* = 34) patients had 1 comorbidity and 16.1% (*n* = 20) had two or more comorbidities. The different signs and symptoms observed in the patients are presented in Table 2. Respiratory symptoms were the most common, these included dyspnea 55.6% (*n* = 69) and cough 64.5% (*n* = 80), followed by systemic symptoms, including fever 52.4% (*n* = 65), fatigue 81.5% (*n* = 101) and osteomuscular pain 70.2% (*n* = 87). Long-term symptoms were frequent (57.3%, *n* = 71), these included common symptoms (39.5%, *n* = 49), respiratory (15.3%, *n* = 19), osteomuscular (8.9%, *n* = 11), neurologic (22.6%, *n* = 28) and psychiatric (19.4%, *n* = 24). Demographic and clinical characteristics in patients with and without long-term COVID-19 symptoms are presented in Table 3.

**Table 1:** Demographic and clinical characteristics of the study population.

**Variable**	**Controls (*n =* 61)**	**Cases (*n =* 63)**	***p*-value**	**CI95%**	**OR**
Age	36.6 (±10.8)	47.3 (±9.53)	<0.01^*^		
Male sex	26 (42.6%)	41 (65.0%)	0.01^*^	1.21–5.18	2.51
**Blood group**					
O	38 (62.3%)	42 (66.7%)	0.61	0.58–2.53	1.21
A	20 (32.8%)	12 (19.0%)	0.08	0.21–1.10	0.48
B	2 (3.3%)	2 (3.2%)	1	0.13–7.09	0.97
AB	1 (1.6%)	0 (0%)	0.98	–	–
**Comorbidities**					
Arrhythmia	0 (0%)	1 (1.58%)	1	–	–
Asthma	2 (3.27%)	1 (1.58%)	0.97	0.04–5.39	0.48
Autoimmune disease	0 (0%)	2 (3.17%)	0.49	–	
Cancer	1 (1.63%)	3 (4.76%)	0.63	0.30–29.66	3.00
Chronic kidney disease	5 (8.2%)	1 (1.58%)	0.22	0.59–45.63	5.17
COPD	0 (0%)	2 (3.2%)	0.49	–	–
Coronary disease	0 (0%)	2 (3.2%)	0.49	–	–
T2DM	1 (1.63%)	13 (20.6%)	<0.01^*^	1.97–123.42	15.6
Hypertension	3 (4.91%)	17 (26.9%)	<0.01^*^	1.97–25.88	7.14
HIV/Immunodeficiency	0 (0%)	2 (3.2%)	0.49	–	–
Obesity	9 (14.7%)	18 (28.5%)	0.09	0.95–5.65	2.31
No comorbidities	47 (77%)	23 (36.5%)	<0.01^*^	0.08–0.38	0.17
One comorbidity	12(19.7%)	22 (34.9%)	0.05	0.97–4.96	2.19
Two or more Comorbidities	2 (3.27%)	18 (28.5%)	<0.01^*^	2.60–53.50	11.80
Chronic use of steroids	1 (1.63%)	1 (1.58%)	1	0.06–15.83	0.97
Smoking history	28 (45.9%)	18 (28.5%)	0.05	0.23–1.02	0.48

* *Statistical significant, p-value < 0.05; COPD, Chronic obstructive pulmonary disease*.

**Table 2:** COVID-19 symptoms in the studied population.

**Variable**	**Controls (*n =* 61)**	**Cases (*n =* 63)**	***p*-value**	**CI 95%**	**OR**
**Acute symptoms**					
Ageusia	40 (65.5%)	23 (36.5%)	<0.01^*^	0.14–0.63	0.30
Anosmia	42 (68.8%)	19 (30.1%)	<0.01^*^	0.09–0.42	0.20
Cough	29 (47.5%)	51 (80.9%)	<0.01^*^	2.10–10.49	4.69
Diarrhoea	11 (18%)	20 (31.7%)	0.07	0.91–4.90	2.11
Dyspnea	13 (21.3%)	56 (88.8%)	<0.01^*^	10.91–80.01	29.54
Fatigue	42 (68.8%)	59 (93.6%)	<0.01^*^	2.12–21.04	6.67
Fever > 38°C	21 (34.4%)	44 (69.8%)	<0.01^*^	2.08–9.38	4.41
Haemoptysis	2 (3.2%)	6 (9.52%)	0.29	0.60–16.03	3.11
Headache	44 (72.1%)	33 (52.3%)	0.02^*^	0.20–0.90	0.42
Mental status disturbance	7 (11.4%)	16 (25.3%)	0.04^*^	1.00–6.93	2.63
Odynophagia	29 (47.5%)	29 (46%)	0.86	0.46–1.91	0.94
Osteomuscular pain	39 (63.9%)	48 (76.1%)	0.13	0.83–3.94	1.81
Rhinorrhea	33 (54%)	24 (38%)	0.07	0.26–1.07	0.52
**Long-term symptoms**					
Presence	26 (42.6%)	45 (71.4%)	<0.01^*^	1.60–7.09	3.37
Common	11 (18%)	38 (60.3%)	<0.01^*^	3.03–15.77	6.91
Cardiovascular	0 (0%)	8 (12.6%)	0.01^*^	–	–
Neurologic	16 (26.2%)	12 (19%)	0.33	0.28–1.55	0.66
Osteomuscular	4 (6.55%)	7 (11.1%)	0.56	0.49–6.42	1.78
Psychiatric	1 (1.63%)	23 (36.5%)	<0.01^*^	4.48–265.78	34.50
Respiratory	4 (6.55%)	15 (23.8%)	0.01^*^	1.39–14.32	4.45
Other long-term symptoms	0 (0%)	2 (3.2%)	0.49		

* *Statistical significant, p-value < 0.05*.

**Table 3:** Demographic and clinical characteristics in patients with and without long-term COVID-19 symptoms.

**Variables**	**Patient with no long-term symptoms (*n =* 53)**	**Patients with long term symptoms (*n =* 71)**	***p*-value**	**CI 95%**	**OR**
Hospitalized	18 (34%)	45 (63.4)	0.00	1.60–7.10	3.37
Age	40 (±12.1)	43.5 (±10.8)	0.095		
Male sex	32 (60.3%)	35 (49.2%)	0.22	0.31–3.22	0.64
**Blood group**					
O	30 (56.6%)	50 (70.4%)	0.11	0.87–3.84	1.83
A	15 (28.3%)	17 (23.9%)	0.58	0.36–1.79	0.80
B	1 (1.88%)	3 (4.22%)	0.82	0.23–22.69	2.29
AB	0 (0%)	1 (1.40%)	1	–	–
**Comorbidities**					
Coronary disease	0 (0%)	2 (2.81%)	0.60	–	–
Arrhythmias	0 (0%)	1 (1.40%)	1	–	–
Hypertension	8 (15.0%)	12 (16.9%)	0.98	0.43–3.03	1.14
COPD	0 (0%)	2 (2.81%)	0.60	–	–
Asthma	1 (1.88%)	2 (2.81%)	0.97	0.04–5.39	0.48
T2DM	6 (11.3%)	8 (11.26%)	1	0.32–3.06	0.99
Chronic kidney disease	4 (7.54%)	2 (2.81%)	0.42	0.06–2.02	0.36
Cancer	1 (1.88%)	3 (4.22%)	0.82	0.23–22.69	2.29
Obesity	10 (18.8%)	17 (23.9%)	0.49	0.56–3.26	1.35
HIV/Immunodeficiency	1 (1.88%)	1 (1.40%)	1	0.05–12.15	0.74
Autoimmune disease	0 (0%)	2 (2.81%)	0.60	–	–
No comorbidities	34 (64.1%)	36 (50.7%)	0.13	0.28–1.19	0.57
One comorbidity	13(24.5%)	21(29.6%)	0.53	0.58–2.90	1.29
Two or more comorbidities	6 (11.3%)	14 (19.7%)	0.31	0.69–5.40	1.92
Chronic use of steroids	0 (0%)	2 (2.81%)	0.60	–	–
Smoking history	14 (26.4%)	32 (45.0%)	0.05	1.03–4.81	2.23
**Acute symptoms**					
Ageusia	20 (37.7%)	43 (60.5%)	0.01^*^	1.22–5.27	2.53
Anosmia	21 (39.6%)	40 (56.3%)	0.07	0.95–4.05	1.97
Cough	31 (58.4%)	49 (69.0%)	0.23	0.75–3.32	1.58
Diarrhoea	10 (18.8%)	21 (29.5%)	0.17	0.77–4.25	1.81
Dyspnoea	18 (33.9%)	51 (71.8%)	<0.01^*^	2.30–10.69	4.96
Fatigue	36 (67.9%)	65 (91%)	<0.01^*^	1.85–14.13	5.12
Fever > 38°C	21 (39.6%)	44 (61.9%)	0.01^*^	1.20–5.15	2.48
Haemoptysis	1 (1.88%)	7 (9.8%)	0.15	0.68–47.72	5.69
Headache	30 (56.6%)	47 (66.1%)	0.28	0.72–3.12	1.50
Odynophagia	23 (43.3%)	35 (49.2%)	0.51	0.62–2.59	1.27
Osteomuscular Pain	32 (60.3%)	55 (77.4%)	0.04^*^	1.03–4.94	2.26
Rhinorrhea	20 (37.7%)	37 (52.1%)	0.11	0.87–3.71	1.80
Brain fog	5 (9.43%)	18 (25.3%)	0.02^*^	1.12–9.46	3.26

* *Statistical significant, p-value < 0.05; COPD Chronic obstructive pulmonary disease*.

### Clinical Association Analysis

Our study revealed a significant statistical correlation between SARS CoV-2 severity and multiple clinical variables reported previously, including age (*p* < 0.01), male sex (*p* = 0.01; OR = 2.51; 95% CI = 1.21–5.18), hypertension (*p* < 0.01; OR = 7.14; 95% CI = 1.97–25.88) and T2DM (*P* < 0.01; OR = 15.6; 95% CI = 1.97–123.42). Interestingly, other clinical variables, including blood group, cardiovascular, pulmonary, and other systemic diseases, such as cancer and obesity, were non-statistically significant in our sample (*p* > 0.05). Additionally, presence of no comorbidities was a protective factor (*p* < 0.01; OR = 0.17; 95% CI = 0.08–0.38) and presence of two or more comorbidities conferred an increased risk of severe disease (*p* < 0.01; OR = 11.8; 95% CI = 2.6–53.5). Symptoms who exhibited significant association with severe disease were mainly respiratory, systemic, and neurological, and included dyspnea (*p* < 0.01, OR = 29.54; 95% CI = 10.91–80.01), cough (*p* < 0.01; OR = 4.69; 95% IC=2.1–10.49) and fever (*p* < 0.01; OR = 4.41; 95% CI = 2.08–9.38) and mental status disturbance (*p* = 0.04; OR = 2.63; 95% CI = 1–6.93). In contrast, anosmia (*p* < 0.01; OR = 0.2; 95% CI = 0.09–0.42), ageusia (*p* < 0.01; OR = 0.30; 95% CI = 0.14–0.63), and headache (*p* = 0.02 OR = 0.42; 95% CI = 0.2–0.9) were more frequent in patients with mild disease (Table 2).

Presence of long-term symptoms was associated with disease severity (*p* < 0.01; OR = 3.37; 95% CI = 1.6–7.1). 42.6% patients in the control group developed these symptoms, in contrast to the 71.4% in the case group. Categories significantly different were common long-term symptoms (*p* < 0.01; OR = 6.91; 95% CI = 3.03–15.77), psychiatric (*p* < 0.01; OR = 34.5; 95% CI = 4.48–265.78) and respiratory (*p* = 0.01; OR = 4.45; 95% CI = 1.39–14.32), whereas cardiovascular long-term symptoms were present only in cases (*p* < 0.01). Multiple acute symptoms were associated with long-term symptoms, such as presence of fatigue (*p* < 0.01; OR = 5.12; 95% CI = 1.85–14.13), osteomuscular pain (0.04; OR = 2.26; 95% CI = 1.03–4.94), dyspnea (*p* < 0.01; OR = 4.96; 95% CI = 2.30–10.69), ageusia (*p* = 0.01; OR = 2.53; 95% CI = 1.22–5.27) and brain fog (*p* = 0.02; OR = 3.26; 95% CI 1.12–9.46).

### Genetic Variants and Association Analysis

The *ACE* rs4646994 genotypic distribution in the total sample was 0.35 (43/124), 0.45 (56/124) and 0.2 (25/124) for Ins/Ins, Ins/Del and Del/Del, respectively. The allele frequency for the Del allele was 0.43 (106/248). For *ACE2* rs2285666, an X-linked SNP, the distribution was 0.5 (29/58), 0.4 (23/58) and 0.1 (6/58) for G/G and G/A and A/A genotypes, respectively, and 0.53 (35/66) and 0.47 (31/66) for G and A genotypes in hemizygous individuals, respectively. The allele frequency for the allele A was 0.36 (66/180). Finally, for *LZTFL1* rs11385942, the distribution was 0.9 (111/124) and 0.1 (13/124) for the genotypes WT/WT and WT/Ins, respectively. We did not observe homozygous individuals for the allele Ins. The allele frequency for this allele was 0.05 (13/235). Genotypic and allelic frequencies are presented in Table 4. All genotypes were found to be in HWE (*ACE* rs4646994 *p* = 0.46, *ACE2* rs2285666 *p* = 0.25 and *LZTFL1* rs11385942 *p* = 1). Genotype frequencies by clinical subgroups (controls, cases hospitalized no ICU and cases hospitalized in ICU) are presented in Supplementary Table 2.

**Table 4:** Allelic and genotypic frequencies for cases and controls.

**Gen**	**SNP**	**Allele frequency controls**		**Allele frequency cases**		**Genotype controls**			**Genotype cases**			**HWE**
		**WT**	**Alt**	**WT**	**Alt**	**WT/WT**	**WT/Alt**	**Alt/Alt**	**WT/WT**	**WT/Alt**	**Alt/Alt**	
*ACE*	rs4646994	0.6	0.4	0.55	0.45	0.39	0.41	0.2	0.3	0.49	0.21	0.46
*ACE2*	rs2285666	0.63	0.37	0.65	0.35	0.47 ♀	0.39 ♀	0.14 ♀	0.55 ♀	0.41 ♀	0.04 ♀	0.25
						0.52 ♂	–	0.48 ♂	0.54 ♂	–	0.46 ♂	
*LZTFL1*	rs11385942	0.98	0.02	0.91	0.09	0.97	0.03	0	0.83	0.17	0	1

*ACE WT allele (Ins), ACE2 WT allele (G), LZTFL WT allele (no dup); Alt, alternative; WT, Wild Type; ♀ Genotype frequencies in females; ♂ Genotype frequences in males (hemizygous)*.

Bivariate analysis between the genetic polymorphisms and COVID-19 severity revealed a statistically significant association between the *LZTFL1* rs11385942 polymorphism with severe COVID-19 and severe COVID-19 requiring hospitalization in ICU (*p* = 0.01; OR = 5.73; 95% CI = 1.24–26.46 and *p* = 0.02; OR = 6.12; 95% CI = 1.14–32.63, respectively, under the allelic genetic model). No association was found between the *ACE* rs4646994 and *ACE2* rs2285666 polymorphisms, and COVID-19 severity under any of the models tested (Table 5). Nevertheless, an association between *ACE* rs4646994 Del and neurological long-term symptoms (e.g., ageusia, anosmia, and vertigo) was identified under the Cochran-Armitage test (*p* < 0.01; OR = 0.32; 95% CI = 0.16–0.63).

**Table 5:** Genetic association analysis for severe COVID-19.

**SNP**	**Model**	**Genotypes/alleles in cases**	**Genotypes/alleles in controls**	**χ2**	**df**	***p-*value**	**OR**	**IC 95%**
ACE rs4646994	Genotypic (2 df) test	13/31/19	12/25/24	1.23	2	0.54	–	–
	Cochran-Armitage trend test	57/69	49/73	0.60	1	0.43	–	–
	Allelic	57/69	49/73	0.65	1	0.41	1.23	0.74–2.03
	Dominant	44/19	37/24	1.15	1	0.28	–	–
	Recessive	13/50	12/49	0.01	1	0.89	–	–
ACE2 rs2285666	Genotypic (2 df) test	1/9/12	5/14/17	–	–	–	–	–
	Cochran-Armitage trend test	11/33	24/48	0.85	1	0.35	–	–
	Allelic	11/33	24/48	0.90	1	0.34	0.92	0.50–1.69
	Dominant	10/12	19/17	–	–	–	–	–
	Recessive	1/21	5/31	–	–	–	–	–
LZTFL1	Genotypic (2 df) test	0/11/52	0/2/59	–	–	–	–	–
rs11385942	Cochran-Armitage trend test	11/115	2/120	6.64	1	<0.01^*^	–	–
	Allelic	11/115	2/120	6.27	1	0.01^*^	5.73	1.24–26.46
	Dominant	11/52	2/59	–	–	–	–	–
	Recessive	0/63	0/61	–	–	–	–	–

* *Statistical significant, p-value < 0.05; df degrees of freedom; Genotypic (2 df) test: Alt/Alt vs. WT/Alt vs. WT/WT; Cochran-Armitage trend test: Alt vs. WT; Allelic: Alt vs. WT; Dominant: Alt/Alt + WT/Alt vs. WT/WT; Recessive: Alt/Alt vs. WT/Alt + WT/WT; ACE WT allele (Ins), ACE2 WT allele (G), LZTFL WT allele (no dup); Alt, alternative; WT, Wild Type*.

### Population Genetic Analysis

Next, we compared the allelic frequencies obtained in this study with those of other datasets including populations of European, Asian, African, North American, and Latin-American ancestries (Supplementary Table 3). We found significant statistical differences for the three systems assessed. For *ACE* rs46469949, East Asia allelic frequencies were the only population with no statistical differences. For *ACE2* the rs2285666 allelic frequency found in our study was similar to those reported in Mexican and American populations. Finally, for *LZTFL1* rs11385942, the comparison was made against COVID-19 patients obtained from a previous study. We found significant differences with Italian controls but not with Italian cases or Spanish population (Table 6).

**Table 6:** Population case-control analysis of allele frequencies.

**SNP**	**Region**	**Total case alleles**	**Cases WT alleles/AF**	**Cases Alt alleles/AF**	***p*-value cases**	**Total controls alleles**	**Controls WT alleles/AF**	**Controls Alt alleles/AF**	***p*-value controls**	**Source**
*LZTFL1* rs11385942	Present study	126	115/0.91	11/0.09		122	120/0.98	20/0.02		
	Italy	1,670	1,436/0.86	234/0.14	0.12	2,510	2,284/0.91	226/0.09	<0.01^*^	(21)
	Spain	1,550	1,410/0.91	140/0.09	0.96	1,900	1,805/0.95	9/0.05	0.14	(21)

* *Statistical significant, p-value < 0.05; AF, Allele frequency; Alt, alternative; WT, Wild Type*.

### Predictive Model and App Development

Genetic and non-genetic significant variables obtained from the previous analyses were entered into a logistic regression model. Different combinations of variables were tested, and the models obtained were compared by Akaike's Information Criterion (AIC) and Coefficient of Discrimination D (Tjur's R2). The best model had the lowest AIC and highest Tjur's R2 values. This model incorporated sex, age, number of comorbidities and the polymorphism *LZTFL1* rs11385942. The resulting predicting score that includes these variables was:


(1) $$\begin{array}{ll} Adjusted\;score\\ = & \frac{1}{1+e^{-\left(-2.88+\left(0.077^{*}age\right)+0.81\left(male\right)+\left(0.99^{*}comorb\right)+1.44\left(WT/Alt\right)\right)}} \end{array}$$

Where the adjusted score is a number between 0 and 1, “*age*” the age in years, “*male*” male sex, “*comorb*” represents the number of comorbidities and “*WT/Alt*” the risk allele for the *LZTFL1* rs11385942 polymorphism.

**Figure 2:**
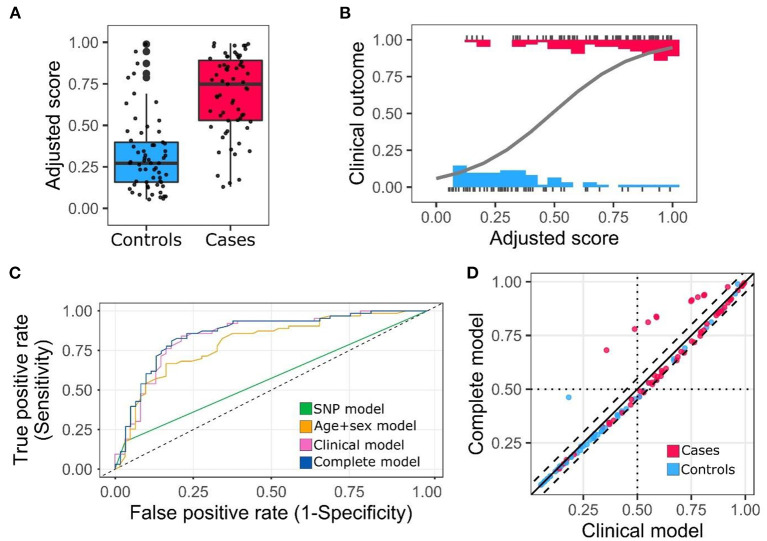
Adjusted score distribution for cases and controls and ROC curve. **(A)** Box plot of the adjusted scores categorized by cases and controls. **(B)** Distribution and regression model for adjusted scores. Clinical outcome 0 corresponds to non-hospitalization and 1 to hospitalization. **(C)** Receiver operating characteristic (ROC) curve. **(D)** Score comparison clinical model vs. complete model. Dashed lines cutoff value ±0.05.

Score distribution using this model for cases and controls is presented in Figures 2A,B. The model achieved good discrimination power (AUC = 0.857; 95% confidence interval 0.79–0.93) (Figure 2C) (Supplementary Table 4). Comparison between the clinical (Age + Sex + Comorbidities) and complete models (Age + Sex + Comorbidities + risk allele) showed a slight increase in the AUC, 0.846 vs. 0.857, respectively. Model comparison was assessed by three additional methods. First, direct comparison between the scores obtained from the clinical and complete model showed a high correlation, nevertheless, for several individuals, the risk scores changed noticeably when the risk allele is included in the model (Figure 2D). Next, we compared the models using the IDI score (52). This method is defined as the difference in the discrimination slopes between two models, the discrimination slopes are calculated as the difference of predicted probabilities for events and non-events (53). We obtained a positive IDI score (0.026; confidence interval 95% 0.001–0.051, *p*-value: 0.039) supporting a significant improvement for the complete model. Third, we calculated cross-validation parameters including concordance, sensitivity, specificity and net benefit for different probability cutoffs (54). Net benefit is a decision analytic measure, which puts benefits and harms on the same scale to be compared (55). The results of this analysis showed that the concordance and net benefit were better for most of the probability cutoffs tested (Supplementary Table 5). This improvement was particularly noticeable at probabilities between 0.3 and 0.4.

Finally, the complete model was used to design a web-based application using the R package Shiny. The application is open-access and is accessible through a shinyApp server (https://oscarortega.shinyapps.io/COVID19_UR_Shiny/). The source code of the shiny app is publicly available on Github at https://github.com/OscarOrt/COVID_19_risk.

## Discussion

During the last 2 years, the COVID-19 pandemic has caused vast disruptions in almost any sphere of human activity. Despite the growing knowledge about the biology and clinical features of this disease, many aspects of its physiopathology and clinical progression remain to be understood. Of particular interest in this process are host risk factors that could contribute to severe courses of COVID-19 and presence of long-term symptoms. These factors include non-genetic and genetic variables. In this study, we aimed to characterize the impact of these variables on COVID-19 outcomes in a sample of Colombian population. We identified several risk factors including the polymorphism *LZTFL1* rs11385942 and incorporated these variables into a predictive model. To the best of our knowledge, this is the first study to evaluate the association between genetic risk factors and COVID-19 severity in a Latin-American population using a case-control design and illustrates the importance of host genetics in SARS-CoV-2 clinical outcomes.

Several non-genetic factors have been associated with poor COVID-19 prognosis, including age, male sex and comorbidities (56). In agreement with such reports, we found a significant association between age, male sex, hypertension and T2DM. Both hypertension and T2DM had been previously identified as independent risk factors for increased morbimortality in COVID-19 patients (57–61). The mechanism by which hypertension is a risk factor has been attributed to hyperactivation of the RAAS pathway, which increases the inflammatory response, cytokine storm, myocardial remodeling, acute lung injury, and endothelial damage (62). Similarly, it has been proposed that T2DM contributes to thromboembolic complications and organ damage through glucotoxicity, oxidative stress, and increased cytokine production (63). Interestingly, hyperglycemia in non-diabetic patients had a negative impact on patient outcomes (64), highlighting the importance of adequate metabolic control in the management of these patients. Other comorbidities analyzed did not show a statistically significant association individually, probably because the sample size was not large enough to detect such associations. Nonetheless, when grouped, the presence of two or more comorbidities conferred an increased risk of severe COVID-19, an effect possibly explained by the additive effect of risk factors to determine the clinical progression of the disease. The second point worth mentioning about clinical features in the studied population was the prevalence of acute symptoms. Among the most common symptoms reported in the literature are generalized weakness, dry cough, headache, dyspnea, and myalgias (65). In our sample, respiratory and systemic symptoms, including dyspnea, cough, fever and fatigue, were associated with severe disease, whereas flu-like symptoms, such as ageusia, anosmia and headache, were more frequent in patients with a mild form of the disease. Other studies, that included, populations have reported similar findings (66, 67). Lower respiratory tract symptoms are often related to severe COVID19, as they are a manifestation of underlying lung compromise.

Another element included in our analysis was the incidence of long-term COVID-19 symptoms, a phenomenon also reported in other viral infections including Spanish Flu SARS CoV-1 and MERS (68). Our findings are consistent with global literature, in which the most common long-term symptoms were fatigue (50–72.8%), joints pain (31.4%), headache (28.9%), chest pain (20–28.9%), dyspnea (28.2%) and palpitations (9%) (68–70). Remarkably, growing evidence suggests that psychiatric illness is an important COVID-19 sequel, affecting particularly specific populations such as Hispanic and African patients (71, 72). Psychiatric long-term symptoms were highly prevalent in hospitalized patients in our study (36.5%). Despite our study being limited by the absence of a standardized mental health scale for patient follow up, our data support these observations (73). The mechanistic basis for these symptoms is attributed to the ability of the virus to infect the central nervous system via the blood-brain barrier and the olfactory bulb, affecting thereafter neurons on the hypothalamus, cortex and brainstem, which could explain many of the neuropsychiatric manifestations (71, 74). On the other hand, the absence of association between comorbidities and long-term symptoms has been also observed in the literature (75). Demographic variables such as sex are of much debate, as there is contradictory evidence of higher rates of long-term symptoms in female individuals (76). Finally, several acute symptoms associated with long-term compromise found in this study have been previously reported in the literature and include fatigue, dyspnea and osteomuscular pain and myalgias (77).

Regarding our genetic findings, our study identified the *LZFTL1* rs11385942 as a significant genetic factor associated with disease severity, conferring risk for severe/critical clinical outcomes. This polymorphism is located in the 3p21.31 locus, a region previously described as an important risk factor for severe respiratory disease in several studies (21, 78). There are six candidate genes in this locus potentially involved in the disease progression presumably by viral entry or clearance and immunological response, these are *SLC6A20, LZTFL1, CCR9, FYCO1, CXCR6*, and *XCR1* (78). The rs11385942 polymorphism is located at intron 5 of *LZFTL1* and recent studies have assessed its functional significance in SARS-CoV-2 infection, suggesting a regulatory role. A CRISPRi analysis using lung epithelial cell lines showed that *LZTFL1* expression is severely affected by this polymorphism (79). LZTFL1 (leucine zipper transcription factor like 1) protein is highly expressed in lung cells and regulates airway cilia and epithelial-mesenchymal transition, a developmental process critical for the innate immune and inflammatory response. Remarkably, the rs11385942 polymorphism has been associated with higher levels of C5a and soluble terminal complement complex C5b-9 (SC5b-9) plasma levels during SARS-CoV-2 infection, suggesting that enhanced immune system and complement activation might be important pathways in the deleterious effect of this variant (80). Moreover, it has been described that complement activation and membrane attack complex (MAC) formation leads to upregulation of pro-inflammatory proteins and inflammasomes causing severe lung injury and, in parallel, endothelial cells death, platelet activation and induction of the coagulation cascade leading to thrombus formation, well-known physiopathological findings in severe COVID-19 (81, 82). The results of another recent study suggest that rs11385942 is in genetic linkage with the polymorphism rs17713054G>A, the gain-of-function risk A allele upregulates the expression of *LZTFL1* by generating a CCAAT/enhancer binding protein beta motif (23). Despite other molecular mechanisms cannot be discarded, this evidence supports *LZTFL1* as a candidate effector and provides further support to our findings. Additional studies have found supporting evidence for this association (79, 83). In line with these observations, genotypification of the risk allele in this gene could be useful as a molecular predictive biomarker for COVID-19 severe/critical clinical outcomes.

Since the beginning of the pandemic, numerous studies have explored the role of host genetic variability in COVID-19 severity and susceptibility. These studies have included genome-wide association studies (GWAS), which have identified multiple reproducible associations (21, 22, 84–86). Given the underrepresentation of Latin American population in these initiatives, our study allowed us to reproduce the association of the 3p21.32 locus in an ethnically different cohort and suggests that the variation in this region modulates the disease outcome (21). Importantly, detailed exploration of “expanded” phenotypes, other than clinical severity, including symptomatic/paucisymptomatic and Exposed_Positive/Exposed_Negative phenotypes have identified a much larger proportion of protective minor alleles (85). These results suggest that using additional phenotype definitions can identify protective associations. Our patients classified as asymptomatic-mild/severe-critical are more likely enriched for risk alleles conferred by loci such as those analyzed in our study.

It is important to highlight that case-control association studies are potentially influenced by population stratification due to undetected population substructure produced by differences in ancestry generating spurious associations (87). To avoid confounding due to population stratification, analysis using ancestry markers (AIMs) are useful to estimate variability between cases and controls (88). Although our study did not carry out this evaluation, we estimate that sampled population shares a similar gene pool without the influence of factors such as geographic isolation or non-random mating. Additionally, the individuals analyzed come from the Colombian Andean region, a geographical area where high inter-individual variation has not been identified (89), which supports the ethnic similarity of the cases and controls included. Here, LZFTL1 rs11385942 was identified as a significant genetic factor associated with severe COVID-19 (*p* = 0.01; OR = 5.73; 95% CI = 1.24–26.46) supporting an important genetic effect. Previously, it has been suggested a need for approaches such as family-based designs or genomic control when the identified genetic effects are very small (OR < 1.20) (90). Finally, although stratification may be less of a concern than originally anticipated and the evidence against a large effect of population stratification, hidden or otherwise, it is important to consider it in false positive or negative association arising from differences in local ancestry (87, 88, 91).

Two polymorphisms analyzed in our study, *ACE* rs4646994 and *ACE2* rs2285666, are important regulators of the RAAS pathway, a physiological system implicated in COVID-19 susceptibility and severity (92). Despite we did not find evidence of association between these polymorphisms and COVID-19 severity, numerous studies support a biological basis for such relationship (92–94). The *ACE2* rs2285666 T allele is associated with a significant increase in ACE2 expression (95). Interestingly, association studies of this polymorphism with COVID-19 severity have had contradictory results and similar findings to ours have been reported by several authors (43, 96, 97). Among these, next-generation sequencing analysis in patients hospitalized for COVID-19 indicated no association between *ACE2* variants and COVID-19 severity (97). Such discrepancies might be explained by population-specific differences, the additive role of other genes interacting with risk alleles or other mechanisms not assessed such as epigenetic modifiers (98–100). Concerning *ACE* rs4646994 the Del allele has been associated with increased ACE expression, higher enzyme activity and elevated production of angiotensin II (101). Despite *ACE* Del/Del genotype and Del allele have been associated with increased COVID-19 patient severity (101–103), our results failed to replicate these findings in the Colombian population. In agreement with our results, other studies have reported no association between *ACE* rs4646994 and COVID-19 severity (43, 96). Collectively, current evidence contains conflicting results about the role of this polymorphism in SARS-CoV-2 infections. The reasons for these discrepancies are unclear and similar to the *ACE2* rs2285666 polymorphism require further exploration. Interestingly, a recent meta-analysis evaluating several polymorphisms related to COVID-19 outcomes found a significant association between the polymorphism ACE rs4646994 and COVID-19 severity (104). Results of individual association studies must be considered carefully and discrepancies in the findings may be the result of underpowered sample sizes, therefore replicates and more robust studies should be considered to validate these associations. On the other hand, we identified *ACE* rs4646994 Del allele as a protective factor for neurological long-term symptoms, we hypothesize this could be related to an increased catalytic activity resulting in vasoconstriction that counterbalances the intracerebral vasodilation and brain edema due to the anaerobic metabolism in cerebral cells in response to SARS-CoV-2 induced hypoxia (105, 106). Whereas, interesting, this hypothesis requires experimental and clinical validation.

Comparison of allelic frequencies obtained in our study with other populations revealed important differences. For *ACE* rs4646994, Asia was the only region with a similar allele frequency to our studied population (40). This may reflect the ancestral origin of Native American population in Colombia or the admixture between an ancestral population with a higher frequency and Europeans, where allele frequencies are considerably lower (107, 108). For the *ACE2* polymorphism, the allelic frequency was similar to Mexican population, probably due to a common ancestry and admixture history (44). For the variant *LZTFL1* rs11385942, no differences were found with Spanish, European and African populations. Remarkably, Zeberg and Pääbo (109) described that the 3p21.31 region, the locus where the variant is located, was inherited from Neanderthals. The mixture of native Americans and Europeans probably modified the ancestral genetic pool leading to the current allele frequencies. Additionally, it has been proposed that differences in allelic frequencies for the 3p21.31 risk haplotype are produced by natural selection in response to pathogens (109).

Another important determinant of COVID-19 severity is viral genetics (10). It has been identified that specific SARS-CoV-2 variants are associated with differences in severity and mortality, for example, the alpha and gamma variants are related to increased hospitalization, ICU admission and mortality risk (110–112). While our study did not assess variant differences in cases and controls, genomic surveillance studies conducted during the sample collection period (December 2020–July 2021) in Bogotá, showed that the predominant variants were B.1.621 (Mu) 57.3% (469/819), P.1 (Gamma) 14% (114/819) and B.1.1.7 (alpha) 2.8% (23/819) (113). The most common variant found in this interval of time, Mu, was classified as a variant being monitored (VBM) by the Centers for Disease Control and Prevention (CDC U.S.) without reported major effects on infectivity, transmissibility or severity (114). The coexistence of several variants during this period constitutes a source of variation and might reflect a more complex dynamics of host-pathogen interactions.

Our clinical and genetic association analysis allowed us to identify several risk factors related to disease severity. These factors were incorporated into a predictive risk model using a multivariate logistic regression including demographic, clinical, and genetic traits. To date, ~50 prediction models and scoring systems, have been published (115). These models are useful tools to facilitate decision-making in healthcare services and rely mostly on clinical features such as age, sex, number of comorbidities, hypertension, T2DM, chronic obstructive lung disease, cancer, cardiovascular disease. However, it is noteworthy that COVID-19 severity is influenced by viral and host genetic factors (10). Recent models, which like ours incorporate a multifactorial approach (genetic and non-genetic factors), included several single nucleotide variants (SNVs) (116). These models have achieved good results in discriminating COVID-19 severity groups and highlighted the role of integrated approaches to predict clinical outcomes. Furthermore, other models aiming to predict adverse outcomes are based on detailed clinical features during diagnosis, admission and hospitalization have been developed, nevertheless, its accessibility and clinical implementation have been limited (117, 118). We propose our model as a useful tool to estimate *a priori* severe or critical illness risk. Notably, despite the minor increase in the AUC when the clinical and complete models were compared, detailed analysis of the discrimination performance and cross-validation parameters suggest that the incorporation of the risk allele improves the risk prediction model. Further studies involving larger sample sizes might be useful to validate these findings. Likewise, the implementation of our model into a web application might facilitate its usage by healthcare providers in limited-resource settings during the current SARS-CoV-2 pandemics and future health emergencies caused by similar pathogens.

In summary, our study explores the relation between non-genetic and genetic factors, with COVID-19 outcomes in Colombian population, demonstrating a positive association between the *LZTFL1* rs11385942 polymorphism and severe disease. By establishing such association, we point up the importance of genetic host factors in SARS-CoV-2 infection. In addition, our work identified previously known non-genetic factors and developed a predictive model which was implemented in a web application, providing a useful tool for risk prediction. Integrative approaches, like ours, may be helpful to better understand COVID-19 clinical progression, refine healthcare efforts and reduce the morbimortality of patients with this disease.

### Study Limitations

Our study has potential limitations. First, the sample size was calculated in order to have 80% statistical power based on previous association reports for the variant with the lower allele frequency (*LZTF1* rs11385942.), nevertheless, it could have been limited to detect potential small effect sizes for the rs4646994 and rs228566 SNPs in our population. Second, some clinical variables assessed in the clinical follow-up interview were self-reported. Even though most of this information was confirmed in the clinical record, this could have been a potential source of bias. Third, we did not match the case-control groups by age or sex for the statistical analysis. Considering these variables are known risk factors, we aimed to assess their impact on COVID-19 outcome. Fourth, as previously mentioned, analysis of potential population stratification was not performed. In addition, COVID-19 severity is a multifactorial trait and other important variables, including environmental factors, SARS-CoV-2 variants, and additional host genetic polymorphisms, described as risk or protective factors were not evaluated. Assessment of such variables in future studies could help to improve discriminative models and medical risk assessment. Finally, we should highlight that our proposed risk model constitutes a proof-of-concept of the feasibility of this integrative approach and further studies with larger sample sizes and independent replications are required to validate the model.

## Data Availability Statement

The original contributions presented in the study are included in the article/Supplementary Material, further inquiries can be directed to the corresponding author/s.

## Ethics Statement

The studies involving human participants were reviewed and approved by Ethics Committee of Universidad del Rosario (DVO005 1543-CV1334). The patients/participants provided their written informed consent to participate in this study.

## Author Contributions

MA-A, DC-O, MG-C, EP-M, CR, DF-M, and OO-R contributed to conception and design of the study. MA-A, PT-F, NC, AM, DF-M, and OO-R performed DNA extraction and genetic analysis. MA-A, DC-O, JC-M, EP-M, CR, PT-F, KP, DF-M, and OO-R collected clinical data and organized the database. MA-A, JC-M, PT-F, and OO-R performed the statistical analysis. MA-A, DC-O, JC-M, PT-F, AM, DF-M, and OO-R wrote sections of the manuscript. All authors contributed to manuscript revision, read, and approved the submitted version.

## Funding

This project was supported by the Universidad del Rosario (Grant IV-TSE026).

## Conflict of Interest

The authors declare that the research was conducted in the absence of any commercial or financial relationships that could be construed as a potential conflict of interest.

## Publisher's Note

All claims expressed in this article are solely those of the authors and do not necessarily represent those of their affiliated organizations, or those of the publisher, the editors and the reviewers. Any product that may be evaluated in this article, or claim that may be made by its manufacturer, is not guaranteed or endorsed by the publisher.
